# Serogroup B Invasive Meningococcal Disease in Older Adults Identified by Genomic Surveillance, England, 2022–2023

**DOI:** 10.3201/eid3005.231714

**Published:** 2024-05

**Authors:** Emily Loud, Stephen A. Clark, David S. Edwards, Elizabeth Knapper, Lynsey Emmett, Shamez Ladhani, Helen Campbell

**Affiliations:** UK Health Security Agency East of England, Harlow, UK (E. Loud, D.S. Edwards, E. Knapper, L. Emmett);; UK Health Security Agency Meningococcal Reference Unit, Manchester, UK (S.A. Clark);; St. George’s University of London, London, UK (S. Ladhani);; UK Health Security Agency, London (S. Ladhani, H. Campbell)

**Keywords:** Neisseria meningitidis, Neisseria meningitidis serogroup B, invasive meningococcal disease, aged, meningococcal infections, genomics, humans, bacteria, meningitis/encephalitis, England

## Abstract

We report a cluster of serogroup B invasive meningococcal disease identified via genomic surveillance in older adults in England and describe the public health responses. Genomic surveillance is critical for supporting public health investigations and detecting the growing threat of serogroup B *Neisseria meningitidis* infections in older adults.

Cases of invasive meningococcal disease (IMD) have declined in England, from 2,595 in 2000 to 205 in 2022 (*1*). During 2021–2022, a total of 89% of IMD cases occurred in persons <25 years of age, and 87% were caused by *Neisseria meningitidis* capsular group B (MenB) strains (*1*). IMD remains a disease of concern, resulting in high rates of disability; the estimated case-fatality rate was 6% during 2021–2022 (*1*). We describe a MenB cluster in older adults in the East of England region and the local and national public health response. The UK Health Security Agency (UKHSA) designated this investigation as a surveillance activity and, thus, ethics review was not required.

## The Study

Case-patient 1 was a 58-year-old woman who experienced a headache and sore throat in early 2023. Her symptoms worsened, she became confused, and she sought care 1 week later at a hospital in East of England, UK (Table). She was intubated, admitted directly to intensive care, and treated with ceftriaxone. *N*. *meningitidis* was cultured from the patient’s blood and cerebrospinal fluid samples. Her case was reported to the local UKHSA team 2 days after hospitalization. Follow-up revealed she had not received any meningococcal vaccinations, nor had she traveled abroad or attended mass gatherings. Two household contacts were given antimicrobial chemoprophylaxis. Case-patient 1 survived.

**Table:** Initial timelines for 2 cases of serogroup B invasive meningococcal disease in older adults identified by genomic surveillance, England, 2022–2023*

Day	Case 1	Case 2
0	Symptom onset	NA
5	Admitted to hospital	NA
6	NA	Symptom onset
7	Case was reported to health protection team; 2 contacts were identified for prophylaxis.	Admitted to hospital
10	NA	Case was reported to health protection team; 1 contact was identified for prophylaxis.
16	UKHSA Meningococcal Reference Unit flagged possible link between cases from 2 isolates.	
17	Epidemiologic link was confirmed, cluster declared, and local partners were alerted. Initial incident management team meeting was held, where cluster was risk assessed and cases were reexamined.	
20	Decision was made to offer vaccination to 9 contacts.	
21	Contacts were informed and vaccinations were arranged with primary care personnel.	

*NA, not applicable; UKHSA, UK Health Security Agency.

Case-patient 2 was an 86-year-old woman who experienced abdominal pain, diarrhea, and vomiting in early 2023. She manifested sepsis at the same hospital 2 days after admission of patient 1 and was treated with amoxicillin/clavulanic acid (Table). *N. meningitidis* was cultured from the patient’s blood sample, and the local UKHSA health protection team was notified 3 days after hospitalization. She had not previously received any meningococcal vaccinations and reported no recent travel history. One household contact was given antimicrobial chemoprophylaxis. Case-patient 2 survived.

Initial contact tracing did not identify any links between the 2 cases. However, on day 16 after patient 1’s symptoms began (Table), the UKHSA’s Meningococcal Reference Unit (MRU) identified both *N. meningitidis* isolates as serogroup B, type 4, subtype P1.12-1,16-183. A review of the records revealed that a contact of patient 1 (who had stayed overnight) had a relative outside of the household who had also been admitted to a hospital. After further investigation, that relative was identified as patient 2. It was then ascertained that this close contact of patient 1 was visiting patient 2 regularly but not staying overnight.

In accordance with UK national public health guidance (*2*), the 3 household contacts were provided antimicrobial chemoprophylaxis and information about IMD. Once the link between the 2 cases was known, an incident management team meeting was convened, involving the health protection team, MRU, local authority public health team, and National Health Service Integrated Care Board members. A decision was made to offer chemoprophylaxis to 3 additional contacts outside of the immediate household. A total of 9 contacts, including the 6 who received prophylaxis and 3 extended family members, were offered the 4CMenB vaccine (Bexsero, https://www.bexsero.com); vaccinations were arranged with each person’s general practitioner. The hospital infection prevention control and microbiology teams were also involved in managing the risks to exposed healthcare workers; some of the healthcare team members involved in the intubation of patient 1 were prescribed antimicrobial chemoprophylaxis.

The MRU identified the isolates from cases 1 and 2 as sequence type (ST) 485 (clonal complex 41/44). Core genome multilocus sequence typing revealed that the 2 isolates clustered closely together in a monophyletic group that had only 4 allelic differences (out of 1422 core genes) between them (Figure) (*3*). Genotypic analysis of meningococcal vaccine antigens showed the isolates harbored factor H binding protein peptide variant 4 and Neisserial heparin binding antigen peptide 2, both of which are expected to cross-react with 4CMenB antibodies (*4*). Therefore, the outbreak strain was predicted to be covered by the 4CMenB vaccine.

**Figure:**
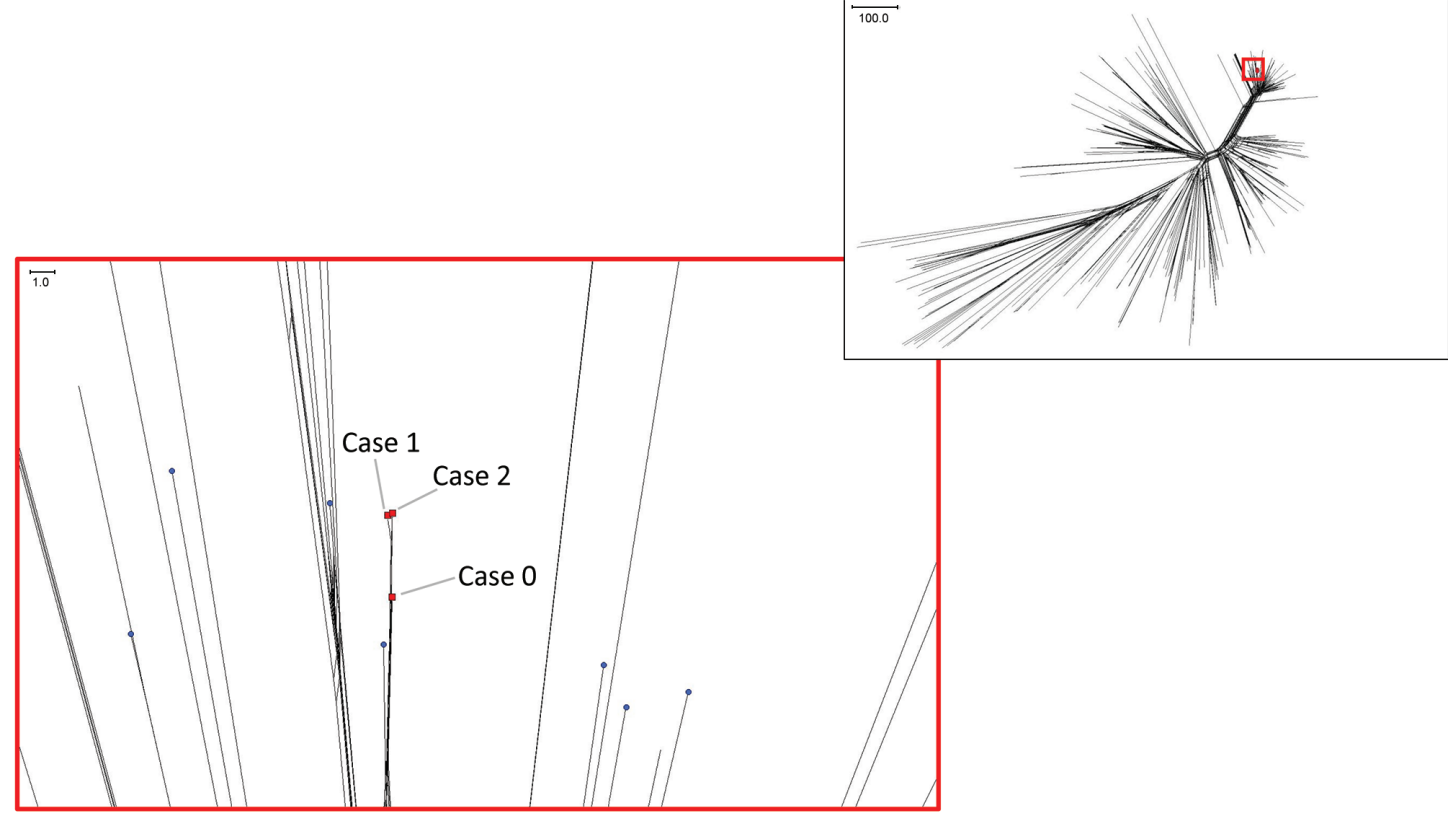
Phylogenetic analysis of *Neisseria meningitidis* isolates in study of serogroup B invasive meningococcal disease in older adults identified by genomic surveillance, England, 2022–2023. Inset indicates the entire phylogenetic tree generated by using the neighbor-net algorithm. Red box indicates the location of the 3 *N. meningitidis* isolates from East of England within the tree. Core genome sequences were compared for *N. meningitidis* clonal complex 41/44 isolates from England, including the 3 East of England isolates, collected during 2016–2023 (n = 356). Comparisons were made by using the Genome Comparator tool (https://www.pubmlst.org). The 3 *N. meningitidis* isolates from East of England clustered closely together in a monophyletic group. Scale bars indicate nucleotide substitutions per site.

Recent cases of ST485 infection have occurred in Yorkshire and the Humber, the Midlands, East of England, and London. An analysis of MenB strain distribution revealed an increasing proportion of MenB cases caused by ST485 since 2010; ST485 was the most common ST among MenB cases in England in 2022 (*5*). An earlier case (designated case-patient 0) within the same area of East of England in autumn 2022 was also caused by MenB ST485. Case-patient 0 was a 92-year-old woman who sought care in late 2022 at the emergency department of the same hospital that admitted patients 1 and 2 (4 months before patient 1’s symptoms began). She had a 3-day history of diarrhea, vomiting, and confusion and died on the same day that she sought care. Her blood culture test was positive for *N. meningitidis*. She had no household contacts or recent travel history. She shared a general practitioner surgery and postal code district with patient 2, but no known contact occurred between the 2 patients. Core genome multilocus sequence typing analysis of the case 0 isolate showed that it clustered closely with the isolates from cases 1 and 2, suggesting that the ST485 strain had persisted in the local area and later caused IMD in patients 1 and 2.

## Conclusions

We identified an unusual cluster of MenB IMD in older adults within a small geographic area (<10 miles across with <20,000 persons) over a 6-month period after an alert from the MRU. Our findings highlight a role for genomic surveillance in supporting standard public health measures and contact tracing processes and enhancing investigations. Furthermore, clinical inquiry and good record keeping during the initial contact tracing process were crucial for identifying the epidemiologic connection between 2 seemingly unrelated cases.

Another unusual feature of this cluster was the patients’ ages. In England, a rapid decline in IMD (along with other infectious diseases) was seen during the COVID-19 pandemic. After removing COVID-19 mitigations in July 2021, an increase in MenB was initially observed among teenagers and young adults, which subsequently expanded across all age groups (S. Clark et al., unpub. data, ). MenB is rare in older adults, but this cluster indicates community transmission of an expanding MenB strain and the ongoing vulnerability of older adults to such strains. Because of increasing vaccination of children and adolescents against IMD in the UK (*6*,*7*), the proportion of cases in older adults will likely grow, a shift that has also been observed across Europe and North America (*8*). This shift is concerning because of the comparatively high case-fatality rates within this older age group (*9*).

Unlike many polysaccharide-conjugated meningococcal vaccines, such as MenC/ACWY, currently licensed MenB vaccines are protein based and do not affect carriage and, therefore, do not confer herd immunity (*10*). Protection against MenB can only be derived from individual vaccination, which in the United Kingdom is only offered to infants born since September 1, 2015 (*11*). Consequently, enhanced strain characterization and surveillance is crucial to provide vaccine strain coverage predictions, which inform decisions on MenB vaccine use during outbreak scenarios (*12*).

In conclusion, although an adolescent MenACWY conjugate vaccine program protects all age groups through herd immunity (*13*), no such protection is conferred for MenB, which could lead to a larger proportion of MenB cases in older adults in the longer term. Limited data exists on meningococcal carriage levels and transmission dynamics in older adults (*14*). Effective strategies, such as genomic surveillance, are needed to prevent and control clusters and outbreaks of *N. meningitidis* infections in this unprotected group of older adults.
